# Strain Construction and Process Development for Efficient Recombinant Production of Mannuronan C-5 Epimerases in *Hansenula polymorpha*

**DOI:** 10.3389/fpls.2022.837891

**Published:** 2022-06-06

**Authors:** Anne Tøndervik, Randi Aune, Adelheid Degelmann, Michael Piontek, Helga Ertesvåg, Gudmund Skjåk-Bræk, Håvard Sletta

**Affiliations:** ^1^Department of Biotechnology and Nanomedicine, SINTEF Industry, Trondheim, Norway; ^2^ARTES Biotechnology GmbH, Langenfeld, Germany; ^3^Department of Biotechnology and Food Sciences, Trondheim, Norway

**Keywords:** *Hansenula polymorpha*, recombinant expression, mannuronan C-5 epimerases, alginate, HCDC

## Abstract

Alginates are linear polysaccharides produced by brown algae and some bacteria and are composed of β-D-mannuronic acid (M) and α-L-guluronic acid (G). Alginate has numerous present and potential future applications within industrial, medical and pharmaceutical areas and G rich alginates are traditionally most valuable and frequently used due to their gelling and viscosifying properties. Mannuronan C-5 epimerases are enzymes converting M to G at the polymer level during the biosynthesis of alginate. The *Azotobacter vinelandii* epimerases AlgE1-AlgE7 share a common structure, containing one or two catalytic A-modules (A), and one to seven regulatory R-modules (R). Despite the structural similarity of the epimerases, they create different M-G patterns in the alginate; AlgE4 (AR) creates strictly alternating MG structures whereas AlgE1 (ARRRAR) and AlgE6 (ARRR) create predominantly G-blocks. These enzymes are therefore promising tools for producing *in vitro* tailor-made alginates. Efficient *in vitro* epimerization of alginates requires availability of recombinantly produced alginate epimerases, and for this purpose the methylotrophic yeast *Hansenula polymorpha* is an attractive host organism. The present study investigates whether *H. polymorpha* is a suitable expression system for future large-scale production of AlgE1, AlgE4, and AlgE6. *H. polymorpha* expression strains were constructed using synthetic genes with reduced repetitive sequences as well as optimized codon usage. High cell density cultivations revealed that the largest epimerases AlgE1 (147 kDa) and AlgE6 (90 kDa) are subject to proteolytic degradation by proteases secreted by the yeast cells. However, degradation could be controlled to a large extent either by co-expression of chaperones or by adjusting cultivation conditions. The smaller AlgE4 (58 kDa) was stable under all tested conditions. The results obtained thus point toward a future potential for using *H. polymorpha* in industrial production of mannuronan C-5 epimerases for *in vitro* tailoring of alginates.

## Introduction

Alginates are linear polysaccharides produced by brown algae and some bacterial species and are composed of the two monomers β-D-mannuronic acid (M) and α-L-guluronic acid (Linker and Jones, 1964; Gorin and Spencer, 1966; Govan et al., 1981; Cote and Krull, 1988). The chemical and physical properties of alginate are determined to a large extent by the sequence and distribution of M and G, which are arranged in consecutive stretches commonly denoted as M-, G-, and MG-blocks in the polymers. Alginate has numerous present and potential future applications within industrial, medical and pharmaceutical areas (Mørch et al., 2007; Hay et al., 2013; Tøndervik et al., 2020; Arlov et al., 2021). G rich alginates are traditionally most valuable and frequently used due to their gelling and viscosifying properties. Mannuronan C-5 epimerases are a family of enzymes responsible for the conversion of M to G at the polymer level during the biosynthesis of alginate (Haug and Larsen, 1969, 1971; Ertesvåg et al., 1995). The alginate producing and nitrogen fixing soil bacterium *Azotobacter vinelandii* has one periplasmic and seven secreted alginate epimerases, all of them involved at some level in biosynthesis of alginate in the complex life cycle of the organism (Ertesvåg et al., 1994, 1995; Svanem et al., 1999; Høidal et al., 2000). The secreted epimerases AlgE1-AlgE7 share a common modular structure, all containing one or two similar but not identical catalytic A-modules (A), and from one to seven similar regulatory R-modules (R). Similar enzymes have been identified in *Azotobacter chroococcum* and in *Pseudomonas syringae* (Bjerkan et al., 2004; Gawin et al., 2020). Intriguingly, despite the structural similarity of the epimerases, they create very different MG patterns in the alginate; AlgE4 being the least complex enzyme (AR) creates alternating MG structure with no G-blocks whereas AlgE1 (ARRRAR) and AlgE6 (ARRR) create predominantly G-blocks, AlgE1 producing longer G-blocks than AlgE6 (Ertesvåg et al., 1995). These different properties make the epimerases very promising tools for producing *in vitro* tailor-made alginates, i.e., alginates that are structurally defined and optimized for specific purposes (Mørch et al., 2007; Tøndervik et al., 2013; Arlov et al., 2014). The epimerases have previously mainly been produced for characterization purposes using *E. coli* as expression host (Ertesvåg et al., 1998; Høidal et al., 1999; Ramstad et al., 1999; Svanem et al., 1999), but alternative bacterial hosts like *Lactococcus lactis* has also been briefly explored (Blatny et al., 2003). Efficient processes for *in vitro* epimerization of alginate requires recombinantly produced alginate epimerases. For this purpose, the methylotrophic yeast *Hansenula polymorpha* is an attractive host organism that has been successfully utilized for expression of several recombinant proteins (Moussa et al., 2012; Manfrao-Netto et al., 2019). The main advantages using this host are the generally stable heterologous expression by chromosomal integration of genes of interest, generally high copy numbers of the integrated genes, lack of endotoxin production and the possibility for efficient secretion of recombinant products into the growth medium facilitating easy recovery from the fermentation broth. Additionally, *H. polymorpha* has status as GRAS (generally regarded as safe) being advantageous from a regulatory point of view. In the present study, our main goal was to investigate whether *H. polymorpha* is a suitable expression system for future large-scale production of the mannuronan C-5 epimerases AlgE1, AlgE4, and AlgE6. To obtain stable expression, strains were constructed using synthetic genes with reduced repetitive sequences as well as optimized codon usage. Cultivation of the epimerase expression strains at high cell densities revealed that the largest epimerases AlgE1 (147 kDa) and AlgE6 (90 kDa) are subject to proteolytic degradation by proteases secreted by the yeast cells, and different strategies for counteracting such degradation was successfully explored.

## Materials and Methods

### Strain Constructions

The genes encoding the epimerases AlgE1, AlgE4, and AlgE6 were assembled by *de novo* chemical synthesis (Geneart, Regensburg), which included adaptation of codon usage as well as reduction of DNA sequence repetitivity in the cases of *algE1* and *algE6*. Importantly, in all cases the amino acid sequence of the enzymes remained unchanged. The synthetic genes (*algE1syn*, *algE6syn*, *algE4syn*) and the *algE1* wild-type gene (*algE1wt*) were each fused in frame to the *Saccharomyces cerevisiae* prepro MFα1 secretion leader sequence and cloned into the expression vector B14 [containing the formate dehydrogenase (FMD) promoter], a derivative of pFPMT121 devoid of antibiotic resistance genes (Klabunde et al., 2007). Expression plasmids were transformed into *H. polymorpha* RB11 (*ura3*) or KLA8-1 (*ura3 leu2*) and uracil-prototrophic transformants were selected. Individual colonies were grown in test tubes containing YPG (2% Bacto peptone/1% yeast extract/2% glycerol/20 mM CaCl_2_) for 2 days at 37°C. Cell-free culture supernatants were screened for epimerase production by dot and Western blotting with an anti-epimerase antibody. The generation of epimerase strains overexpressing *HpCNE1* was described previously (Klabunde et al., 2007). Overexpression of both *HpCNE1* and *HpSEB1* was achieved in a similar fashion by introduction of a plasmid containing both chaperone genes. Protease knockout mutants (*yps1*, *yps7*, *pep4*, and 3 additional genes predicted to encode proteases) were generated in strain KLA8-1 by targeted deletion using *S. cerevisiae LEU2* as the selectable marker.

### High Cell Density Fermentations

The fermentation media used was YSPG (25 g/l glucose, 10 g/l yeast extract, 20 g/l soyton pepton, 55 mM CaCl_2_) or HCD (10.9 g/l KH_2_PO_4_, 5.8 g/l (NH_4_)_2_HPO_4_, 5 g/l (NH_4_)_2_SO_4_, 2.3 g/l KCl, 0.5 g/l NaCl, 0.1 g/l Na-EDTA, 25 g/l glucose, 25 g/l yeast extract, 18 mM MgSO_4_, 55 mM CaCl_2_) or combinations of these as described in the text. For both media, 20 ml/l of a vitamin solution (thiamine-HCl 5 g/l, biotin 0.015 g/l) and 10 ml/l of a trace mineral solution (H_3_BO_3_ 0.05 g/l, (NH_4_)_2_Fe(SO_4_)_2_ ⋅ 6H_2_O 10 g/l, CuSO_4_ ⋅ 5H_2_O 0.8 g/l, ZnSO_4_ ⋅ 7H_2_O 3 g/l, MnSO4 ⋅ H_2_O 4 g/l, NiCl_2_ ⋅ 6H_2_O 0.09 g/l, CoCl_2_ ⋅ 6H_2_O 0.1 g/l, Na_2_MoO_4_ ⋅ 2H_2_O 0.1 g/l, KI 0.1 g/l, HCl 50 ml/l) was added. If not otherwise stated, the cultivation temperature used was 37°C, and pH in YSPG and HCD was 6.0 and 5.5, respectively. The feeding solution used was glucose (30 or 60%) with methanol (10% of glucose). The feeding was started at a low rate (3–5 g/L, h) approx.12 h after inoculation and increased to 5–7 g/L, h after approx. 20 h or when the cultures were glucose limited. The rest of the fermentation was then run with glucose limitation. Precultures for fermentations were grown in shake-flasks in a medium containing 5 g/l yeast extract and 20 g/l soytone peptone for 16–18 h before inoculation. At the end of a standard 4-day fermentation process, samples from the cultures were routinely checked for purity by plating on solid medium with the same composition as for the pre-cultivation.

### Determination of Epimerase Activity

For determination of epimerase activity, a spectrophotometric assay was developed using mannuronan (polyM) isolated from a mutant strain of *Pseudomonas fluorescens* NCIMB 10525 as substrate (Gimmestad et al., 2003). Dilutions of epimerase-containing samples, i.e., culture supernatants were mixed with polyM (1 mg/ml) in a buffer composed of 20 mM MOPS, 3.6 mM CaCl_2_ and 100 mM NaCl and incubated at 37°C for 16–18 h. Alginate lyase AlyA degrading G-M and G-G linkages (Tøndervik et al., 2010) was then added to a final concentration of 0.05 U/ml. Incubation was continued at 25°C for 4 h with recording of absorbance at 230 nm (A_230_) before (T_0_) the addition of AlyA and after 4 h (T_1_) and subtracting the values of a buffer blank. Lyase degradation introduces unsaturated uronic acid residues that can be quantified by A_230_, and the epimerase activity (i.e., introduction of G residues into polyM) in the samples is thus proportional to the ΔA_230_ (T_1_-T_0_) obtained. One unit of epimerase activity is defined as the amount of enzyme resulting in ΔA_230_ = 1 under the described conditions.

### NMR Analysis of Epimerized Alginate Samples

All experiments were recorded on a BRUKER Avance 600 or DPX 400 spectrometer equipped with a 5 mm cryogenic CP-TCI z-gradient probe and 5 mm z-gradient DUL (C/H) probe, respectively. End-point analysis of epimerized samples were recorded at 363 K. To reduce the viscosity of the alginate samples prior to NMR measurements the samples were depolymerized by mild acid hydrolysis to a final average DP_*n*_ ∼30 residues. 3-(trimethylsilyl)-propionic-2,2,3,3-d4 acid sodium salt (Aldrich, Milwaukee, WI, United States) was used as internal standard for the chemical shift and triethylenetetra-amine hexa-acetate (Sigma-Aldrich) was added to chelate residual calcium ions in end-point epimerized samples.

### Cell Sampling, Isolation of RNA and RT-PCR Analysis

Yeast cells from fermentations were harvested by centrifugation (14,000 rpm, 5 min), supernatants were removed, and pellets frozen immediately in liquid nitrogen before storage at −80°C. RNA was isolated from yeast cells by the RNeasy Mini Kit (QIAGEN) applying mechanical disruption for lysis of cells (0.5 mm glassbeads and MiniBeadBeater). RNA concentration was measured by a NanoDrop Spectrophotometer ND-1000. Potential residual DNA was removed using the DNA-*free*™ kit from Ambion, and cDNA synthesis was performed using the First-Strand cDNA Synthesis Kit from GE Healthcare. The quantitative PCR was performed using Power SYBR^®^ Green and a 7500 Real Time PCR System from Applied Biosystems. The PCR profile was as follows: stage 1; 50°C, 2 min (1 cycle) stage 2; 95°C, 10 min (1 cycle) stage 3; 95°C, 15 s and 60°C, 1 min (40 cycles). The actin (*act*) gene of *H. polymorpha* was used as an endogenous control. The primers used are listed in Table 1. All PCR reactions were performed in triplicates.

**TABLE 1:** Primers used for gene expression analyses of algE1syn and algE4syn.

Primer	Sequence 5′→ 3′
algE1syn-fwd	GCTGGAGGAGGTACTGTGTACTTG
algE1syn-rev	TGGCGACACCCTGTATTCAC
algE4syn-fwd	GATGACCAACAACGTGGCTTAC
algE4syn-rev	CTGCACAACCAGTCCAGAAGAA
act-fwd	GACCTTCAACGTGCCAGCAT
act-rev	AACGACAGCACAGCCTGGAT

### Other Methods

Deglycosylation of proteins in the culture supernatants before SDS-PAGE analysis was performed by adding Endoglucosidase H (Roche) to supernatant samples (1:45) and incubating at 37°C overnight. N-terminal sequencing of protein fragments from the *H. polymorpha* supernatant was performed by separating the proteins by SDS-PAGE followed by blotting them on PVDF membranes as described earlier (Ertesvåg et al., 1994). The sequencing was performed by the Biotechnology Centre of Oslo at the University of Oslo. AlgE1 and the shortened derivatives used for the spiking experiments were expressed and purified as previously described (Ertesvåg et al., 1998).

## Results and Discussion

### Generation and Initial Characterization of *Hansenula polymorpha* Strains for Expression of Alginate Epimerases AlgE1, AlgE4, and AlgE6

**FIGURE 1:**
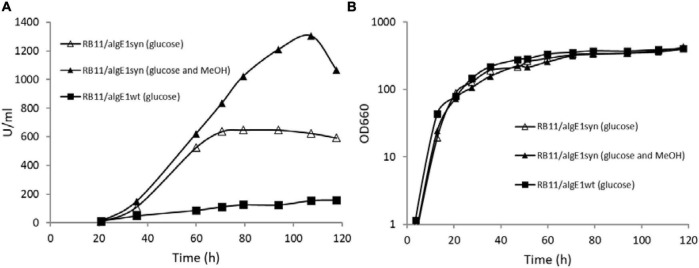
AlgE1 epimerase activity (U/ml) produced **(A)** and growth **(B)** of RB11/algE1wt and RB11/algE1syn when grown in fermentors with HCD medium (pH 4.6, 37°C) and 60% glucose or glucose (60%) and methanol (10% of glucose) in the feeding solution.

**FIGURE 2:**
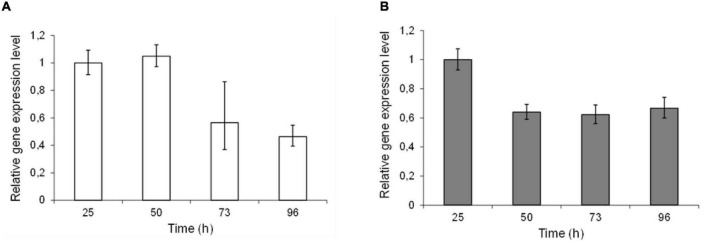
Relative expression levels of epimerase genes in RB11/algE1syn **(A)** and RB11/algE4syn **(B)** during cultivation in HCD medium with feeding solution composed of glucose (60%) and methanol (10% of glucose). Samples for DNA isolation were taken during the fermentation at 27, 50, 73, and 96 h after inoculation. For each strain the expression level in sample 1 is set as 1. Quantifications were performed in triplicate and standard deviations from the mean are indicated by error bars.

Epimerase production, secretion and activity was first analyzed in small-scale test-tube cultures. In the case of AlgE1, the activity was fully dependent on the presence of excess calcium ions in the growth medium. Furthermore, it was found that only about 10% of the strains transformed with wild type *algE1* and screened (approx. 100 transformant cultures) for epimerase activity secreted an active enzyme. The native gene contains several repetitive sequences, and the GC content is high. A striking improvement of AlgE1 production, both in terms of the frequency of expression strains obtained and the level of secreted epimerase, resulted from introduction of a synthetic gene (*algE1syn*) with less repetitive sequences and a codon usage adapted to *H. polymorpha.* By comparing the production of AlgE1 from strains expressing wild type (RB11/algE1wt) and synthetic gene (RB11/algE1syn) during fermentation in HCD medium, we found that the epimerase activity detected in the culture medium (U/ml) increased about fourfold by the introduction of an optimized gene (Figure 1A), proving the importance of codon adaptation. Furthermore, it was also found that addition of methanol to the feeding solution (to 10% of the glucose concentration used) led to another twofold increase in the secreted epimerase activity, presumably due to induction of expression from the FMD-promoter under the glucose limiting conditions (Figure 1A). The two strains expressing either wild type or synthetic *algE1* showed identical growth kinetics and growth yield in the medium utilized, and these parameters were not influenced by addition of methanol (Figure 1B). In all further fermentation experiments we therefore routinely used methanol in the feeding solution if not stated otherwise. Based on the experiences with expression of *algE1* in *H. polymorpha*, strains for expressing *algE4* and *algE6* were constructed using synthetic genes only (see Supplementary File 1 for alignments of wt and synthetic genes for *algE1*, *algE4*, and *algE6*). As described below, stability of AlgE1 is a significant issue using *H. polymorpha* as expression host. To elucidate whether expression of the integrated epimerase genes could be a bottleneck during the fermentations, expression analysis of *algE1* and *algE4* were performed by RT-PCR using the *H. polymorpha* actin gene (*act*) as an endogenous control. The strains tested were RB11/algE1syn and KLA8-1/algE4syn. Fermentations were performed using HCD medium at 37°C, and samples for isolation of RNA and production analysis were taken at 27, 50, 73, and 96 h after inoculation. The expression level of the genes was found to be quite stable during the cultivation period (Figures 2A,B), however with a higher level at the two first timepoints for RB11/algE1syn and the first timepoint for KLA8-1/algE4syn. These data indicate that the main challenges connected to AlgE1 production is related to stability of the produced protein.

### Recombinant Alginate Epimerase AlgE1 Is Subject to Degradation by Host Proteases During Fermentation

**FIGURE 3:**
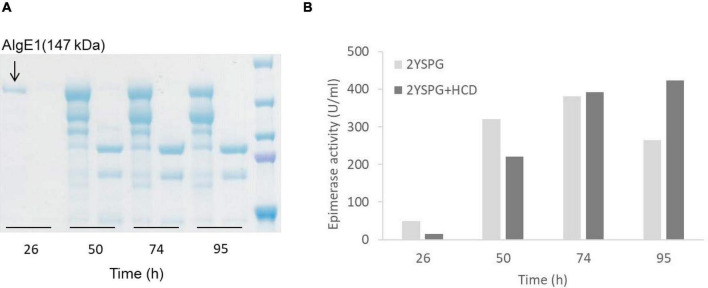
SDS-PAGE analysis of AlgE1 stability during fermentation of RB11/algE1syn in 2xYSPG (37°C, pH 6) and 2xYSPG + 1/3HCD (37°C, pH 6) both with feeding solutions composed of glucose (30%) and methanol (10% of glucose) in samples taken at 26, 50, 74, and 95 h **(A)**, and AlgE1 epimerase activity (U/ml culture) of the same samples **(B)**. The samples on the SDS-PAGE gel are arranged in the same order as in the bar chart shown in panel B of the figure. Size of protein fragments in standard (right); 250, 150 100, 75, and 50 kDa.

Epimerase producing *H. polymorpha* strains were initially fermented in HCD medium, and for determining the yield and integrity of the secreted enzymes, samples taken during fermentation were analyzed by protein gel-electrophoresis (SDS-PAGE). Through these analyses it became evident that all three epimerases were heavily N-glycosylated, however glycosylation did not measurably compromise the enzymatic activity (data not shown). It was also found that AlgE1 (147 kDa) was degraded into two main smaller fragments during the fermentation process, and only very faint signals corresponding to full length AlgE1 were visible on the gel (data not shown). Degradation could be partly controlled by growing the strains in a less rich medium (2xYSPG), Figure 3A. However, when a fraction of HCD (to a concentration of 1/3) was added to the medium attempting to increase the biomass yield, this again led to severe degradation. From the SDS-PAGE analyses it appears that degradation is taking place during the entire fermentation period, indicating that this is caused by one or several proteases that are secreted by the yeast cells independently of the growth phase.

**FIGURE 4:**
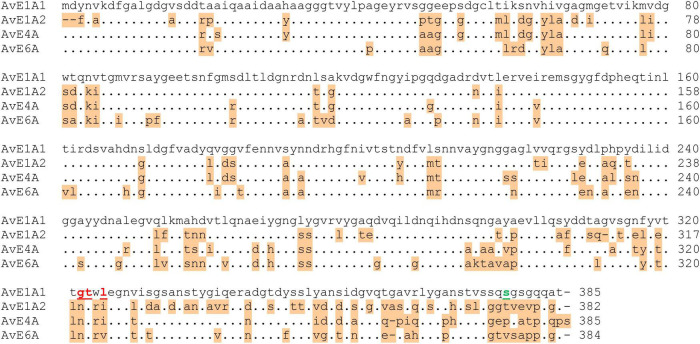
Alignment of the A-modules of AlgE1, AlgE4, and AlgE6 emphasizing the differences between the first A module of AlgE1 (A1) and the other three A-modules. Dots indicate identical amino acids whereas amino acids differing are shaded. The amino acid identified as the N-terminal in the degraded AlgE1 produced by *H. polymorpha* is shown in green, while the ones identified by incubation of supernatant from *H. polymorpha* with purified AlgE1 is marked in red.

Despite substantial degradation, epimerase activity was still detected in the supernatants indicating that the degraded enzyme retained activity (Figure 3B). NMR analysis of mannuronan epimerized with degraded enzyme partially purified from the supernatant showed that the alginate obtained did not contain a high level of G-blocks as is the case for intact AlgE1. The level of G-blocks decreased during the fermentation with and accompanied increase in the level of alternating MG/GM residues (Table 2). This indicated that the residual activity could be caused by an intact A2 module which introduces single G’s and degradation of the A1 module of AlgE1 which is responsible for the block forming properties (Ertesvåg et al., 1998). This was confirmed by obtaining the N-terminal sequence of the protein fragment with the highest Mw (confer Figure 3A). The sequence SGSGQ indicated that the protein was cut after residue 371 in the A1 module of AlgE1 (Figure 4). The remaining part contains all R modules and it has previously been shown that proteins that contain at least the last R module and the second A module of AlgE1 have a specific activity not much less than the wild-type enzyme (Ertesvåg et al., 1998).

**TABLE 2:** NMR analyses of polyM epimerized with AlgE1-containing samples from fermentations of RB11/algE1syn showed in Figure 1.

Sample	F_*G*_	F_*M*_	F_*GG*_	F _*MG/FGM*_
RB11/algE1syn (glucose, MeOH), 35 h	0.44	0.56	0.13	0.31
RB11/algE1syn (glucose, MeOH), 110 h	0.44	0.56	0.07	0.38
RB11/algE1syn (glucose), 35 h	0.49	0.51	0.22	0.27
RB11/algE1syn (glucose), 110 h	0.44	0.56	0.06	0.38

*F_G_ and F_M_ denote the fractions of mannuronan and guluronan in the epimerized alginate, and F_GG_ and F _MG/GM_ the fractions of consecutive GG and MG or GM residues.*

### Exploring the Use of Protease Deficient Strains to Avoid Epimerase Degradation

In the case of extracellular proteases, it should be possible to use the supernatant from *H. polymorpha* known to degrade the produced AlgE1 to degrade purified proteins containing only one of the two A-modules of AlgE1 expressed from individual vectors (Ertesvåg et al., 1998). SDS-PAGE showed that the proteins containing the A1 module was degraded into smaller fragments, whereas the A2 module remained intact (not shown). The protein fragments from the A1 module were subjected to N-terminal sequencing, which indicated that the protein firstly is cut close to the end of the A-module (Figure 4). Prolonged incubation resulted in a band with smaller molecular weight consisting of several peptides that could not be resolved by N-terminal sequencing. Based on the effect of protease inhibitors, it seemed probable that more than one protease is involved in the degradation. Heat inactivation of the supernatant by boiling prior to incubation with purified AlgE1 abolished degradation, indeed pointing to an enzymatic degradation reaction, and attempts were therefore made to identify the protease(s).

This information could potentially be used for specific gene inactivation of the relevant targets. The supernatant from *H. polymorpha* known to degrade AlgE1 was fractionated using a gel-filtration column, and fractions displaying proteolytic activity were separated on SDS-PAGE before transferring proteins to PVDF membranes by western blotting. Proteins were visualized with Coomassie staining and bands excised from the membrane for analysis with MS after trypsination. *H. polymorpha* proteins were identified by utilizing genome/protein data (Ramezani-Rad et al., 2003), but unfortunately none were recognized as potential proteases. Presumably, the relevant proteases exist in very minor amounts, and there might also be several responsible proteases acting sequentially as vulnerable parts of the epimerase are gradually being exposed to the environment.

Several cellular proteases have previously been found to contribute to degradation of secreted heterologous proteins in yeast. These include proteases of the yapsin (Yps) family and the vacuolar proteinase A (Pep4). We generated mutants in strain KLA8-1 that lacked the *H. polymorpha* homologs of the genes *YPS1*, *YPS7*, *PEP4*, and three other genes predicted to encode proteases from the genome annotation data (Ramezani-Rad et al., 2003). The effect of the knockouts on epimerase stability was investigated by spiking the fermenter broth in which individual mutants had been grown, or cell suspensions, with purified *E. coli*-derived AlgE1. However, none of the protease knockouts significantly enhanced the stability of AlgE1 (data not shown). The KLA8-1 protease deficient strains constructed can be of value for similar degradation susceptibility evaluations in future studies.

### Increased Calnexin Gene Dosage Increases Secretion of AlgE1 and AlgE4

**FIGURE 5:**
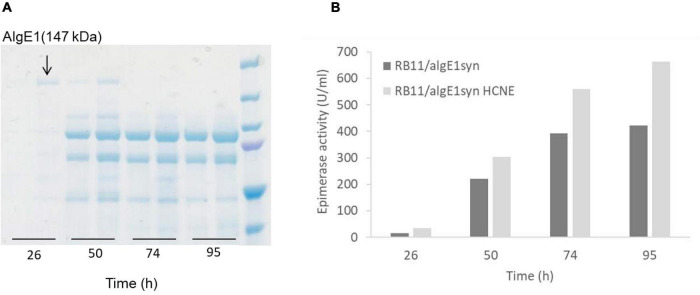
Effect of increased calnexin gene dosage (*HpCNE*) on AlgE1 stability analyzed by SDS-PAGE **(A)** and epimerase activity (U/ml culture) **(B)** in RB11/algE1syn and RB11/algE1syn HCNE. Strains were cultivated in 2xYSPG + 1/3HCD (37°C, pH 6) with feeding solution composed of glucose (60%) and methanol (10% of glucose), and samples were taken at 26, 50, 74, and 95 h. The samples on the SDS-PAGE gel are arranged in the same order as in the bar chart shown in panel B of the figure. Size of protein fragments in standard (right); 250, 150 100, 75, 50, and 37 kDa.

It has previously been shown that increased gene dosage of the molecular chaperone calnexin (*HpCNE1*) enhances secretion of AlgE1 in recombinant *H. polymorpha* production strains when analyzed by cultivation in shake flasks (Klabunde et al., 2007). Here, we show that this is also the case when strains are grown to high cell density in fermentors, resulting in an increased epimerase activity at the end of a standard 4-day fermentation experiment (Figure 5B). However, overexpression of calnexin did not protect the produced AlgE1 from proteolytic degradation, indicating that the host proteases still have access to specific target sites in the protein (Figure 5A).

**FIGURE 6:**
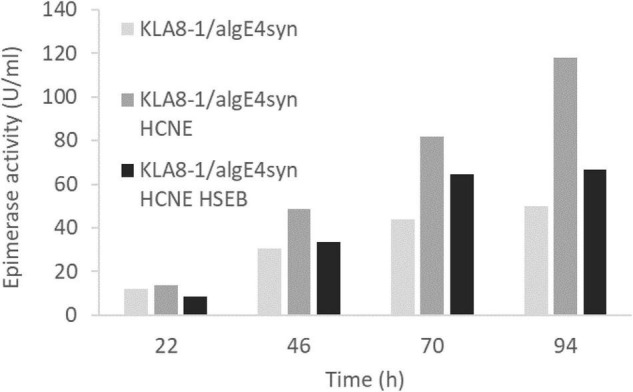
Effect of increased calnexin (HpCNE) and SEB1 (HpSEB) gene dosage on the production of AlgE4 in *H. polymorpha*. Specific AlgE4 epimerase activity (U/ml culture) obtained with KLA8-1/algE4syn, KLA8-1/algE4syn HCNE, and KLA8-1/algE4syn HCNE HSEB during the fermentation process with samples taken at 22, 46, 70, and 94 h. Strains were cultivated in HCD (37°C, pH 5.5) and with feeding solutions composed of glucose (60%) and methanol (10% of glucose).

Contrary to AlgE1, we found that the smaller AlgE4 was not subject to degradation when expressed in *H. polymorpha*, presumably due to absence of the main target amino acid sequence of protease attack identified in AlgE1 (Figure 4). The effect of calnexin dosage on expression on AlgE4 was also explored. Strain KLA8-1/algE4syn HCNE was constructed and found to give approximately twofold increase in the secreted AlgE4 activity compared to the parental strain KLA8-1/algE4syn (Figure 6). Moreover, in order to attempt strengthening the protein folding apparatus further, additional copies of the *H. polymorpha* SEB1 ortholog (*HSEB1*) were inserted into KLA8-1/algE4syn HCNE. Overexpression of this subunit of the SEC61 translocon complex has previously been shown to increase secretion of certain heterologous proteins (Boisrame et al., 2002; Toikkanen et al., 2004). However, the expression of *HSEB1* apparently counteracted the positive effect exerted by increased calnexin dosage since the specific AlgE4 activity obtained with KLA8-1/algE4syn HCNE HSEB was reduced compared to KLA8-1/algE4syn HCNE (Figure 6). The reason for this is not clear but might be connected to overloading the total protein expression capacity of the *H. polymorpha* cells.

### Growth on Glycerol Increases the Stability of AlgE6

**FIGURE 7:**
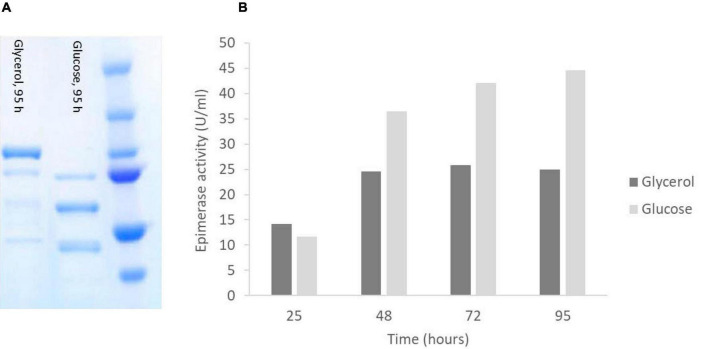
SDS-PAGE analysis **(A)** and epimerase activity (U/ml culture) **(B)** of RB11/algE6syn cultivated in 2xYSPG with glycerol in the basis medium and a feeding solution composed of 60% glycerol, or in 2xYSPG + 1/3 HCD (glucose in basis medium) and feeding solution composed of glucose (60%) and methanol (10% of glucose). Samples for activity analysis was taken at 25, 48, 72, and 95 h. SDS-PAGE show the AlgE6 content in the samples taken at 95 h. Size of protein fragments in standard (right); 250, 150 100, 75, 50, and 37 kDa.

As with AlgE1, it was found that AlgE6 was degraded to a large extent during the fermentations when strain RB11/algE6syn was cultivated in HCD and in 2xYSPG + 1/3HCD with glucose and methanol in the feeding solution. Basically, no full-length AlgE6 was visible on the gels during the fermentation period (Figure 7A). In the initial strain characterizations carried out in small scale shake-flasks in a medium containing yeast extract, peptone and glycerol stability was however not a problem. Fermentation experiments were therefore performed with 2xYSPG where glycerol was used as a carbon source instead of glucose in the basis medium, and with 60% glycerol as feeding solution. Growth in glycerol medium resulted in significantly lower biomass, i.e., at the end of the cultivation (95 h) OD_660_ was approx. 80, whereas with glucose the corresponding value was 250. Due to the lower cell mass the activity per ml detected was lower (Figure 7B), however the AlgE6 produced remained mainly intact (Figure 7A).

Despite the degradation observed when RB11/algE6syn was fermented in glucose-based medium (2YSPG + 1/3HCD), epimerase activity was detected. This might indicate that the catalytic A-module of AlgE6 is not affected by proteolytic degradation, rather the three C-terminal non-catalytic R-modules are the protease target. The A module of AlgE6 has previously been shown to be active without its associated R-modules (Stanisci et al., 2018). The degradation pattern of AlgE6 was, however, not investigated in more detail. The results obtained with AlgE6 show that by modifying the composition of the cultivation medium it is possible to increase the stability of the epimerase product. Due to the similarity of AlgE6 and AlgE1, these findings will probably also apply equally well to AlgE1, but this needs to be evaluated in future studies.

Data from production of the epimerases by high cell density cultivations of *E. coli* (OD of approx. 200) show that for AlgE1 higher levels in terms of U/ml culture are obtained in *H. polymorpha*, whereas for AlgE4 and AlgE6 higher levels are obtained in *E. coli* (unpublished). Furthermore, in *E. coli* the epimerases are not subject to degradation.

## Conclusion

The potential for using the yeast *H. polymorpha* for expression of mannuronan C-5 epimerases AlgE1, AlgE4, and AlgE6 was investigated. For construction of epimerase expression strains, synthetic genes were used since wild type genes with several direct repeats, were found to be highly unstable when integrated into the yeast chromosome. Fermentation of the epimerase-expressing strains under conditions giving high cell densities (OD_660_ > 250) resulted in degradation of AlgE1 and AlgE6, but not AlgE4. AlgE1 which consists of two catalytic A-modules each giving rise to fundamentally different epimerase patterns, was found to be fragmented by cleavage of the N-terminal A1 module, while leaving the A2 module intact and active. Degradation due to host proteases secreted into the medium could be reduced but not avoided for AlgE1 by co-expressing the chaperone protein calnexin. Co-expression of calnexin in AlgE4 producing strains increased the epimerase activity obtained per cell mass, indicating that although not subject to proteolytic degradation AlgE4 stability and activity can be improved by this strategy. Increased stability of AlgE6 was obtained by using glycerol in the feeding medium instead of glucose. Although the secreted epimerases were not purified from the culture supernatants, it could be observed from the SDS-PAGE gels that the recombinant proteins were dominating. Compared to intracellular expression in *E. coli* which necessitates cell disruption before purification, the down-stream processing from secretory production in *H. polymorpha* would be simpler. Altogether, the results obtained show that using *H. polymorpha* for epimerase expression involves challenges related to both the DNA sequence as well as the properties of the recombinant enzymes. However, strategies targeted both to the level of strain construction and process development show that these challenges can be met and large-scale production of epimerases in this GRAS organism are therefore considered as a promising approach.

## Data Availability Statement

The raw data supporting the conclusions of this article will be made available by the authors, without undue reservation.

## Author Contributions

AD and MP performed the strain construction and initial characterization. HS and RA performed the fermentation experiments. AT and HE performed the analytical work. AT wrote the first draft of the manuscript. All authors reviewed, edited the manuscript, contributed to the planning, and design of experiments.

## Conflict of Interest

AD and MP were employed by the ARTES Biotechnology GmbH. The remaining authors declare that the research was conducted in the absence of any commercial or financial relationships that could be construed as a potential conflict of interest.

## Publisher’s Note

All claims expressed in this article are solely those of the authors and do not necessarily represent those of their affiliated organizations, or those of the publisher, the editors and the reviewers. Any product that may be evaluated in this article, or claim that may be made by its manufacturer, is not guaranteed or endorsed by the publisher.

## References

[B1] Arlov O. Aachmann F. L. Sundan A. Espevik T. Skjåk-Bræk G. (2014). Heparin-like properties of sulfated alginates with defined sequences and sulfation degrees. *Biomacromolecules* 15 2744–2750. 10.1021/bm500602w 24844124

[B2] Arlov O. Rutsche D. Korayem M. A. Ozturk E. Zenobi-Wong M. (2021). Engineered sulfated polysaccharides for biomedical applications. *Adv. Funct. Mat.* 31:52.

[B3] Bjerkan T. M. Bender C. L. Ertesvåg H. Drabløs F. Fakhr M. K. Preston L. A. (2004). The *Pseudomonas syringae* genome encodes a combined mannuronan C-5-epimerase and O-acetylhydrolase, which strongly enhances the predicted gel-forming properties of alginates. *J. Biol. Chem.* 279 28920–28929. 10.1074/jbc.M313293200 15123694

[B4] Blatny J. M. Ertesvåg H. Nes I. F. Valla S. (2003). Heterologous gene expression in *Lactococcus lactis*; expression of the *Azotobacter vinelandii* AlgE6 gene product displaying mannuronan C-5 epimerase activity. *FEMS. Microbiol. Lett.* 227 229–235. 10.1016/S0378-1097(03)00685-2 14592713

[B5] Boisrame A. Chasles M. Babour A. Beckerich J. M. Gaillardin C. (2002). Sbh1p, a subunit of the Sec61 translocon, interacts with the chaperone calnexin in the yeast *Yarrowia lipolytica*. *J. Cell Sci.* 115 4947–4956. 10.1242/jcs.00187 12432081

[B6] Cote G. L. Krull L. H. (1988). Characterization of the exocellular polysaccharides from *Azotobacter chroococcum*. *Carbohyd. Res.* 181 143–152. 10.1016/0008-6215(88)84030-8

[B7] Ertesvåg H. Doseth B. Larsen B. Skjåk-Bræk G. Valla S. (1994). Cloning and expression of an *Azotobacter vinelandii* mannuronan C-5 epimerase gene. *J. Bacteriol.* 176 2846–2853. 10.1128/jb.176.10.2846-2853.1994 8188585 PMC205438

[B8] Ertesvåg H. Høidal H. K. Hals I. K. Rian A. Doseth B. Valla S. (1995). A family of modular type mannuronan C-5 epimerase genes controls alginate structure in *Azotobacter vinelandii*. *Mol. Microbiol.* 16 719–731. 10.1111/j.1365-2958.1995.tb02433.x 7476166

[B9] Ertesvåg H. Høidal H. K. Skjåk-Bræk G. Valla S. (1998). The *Azotobacter vinelandii* mannuronan C-5-epimerase AlgE1 consists of two separate catalytic domains. *J. Biol. Chem.* 273 30927–30932. 10.1074/jbc.273.47.30927 9812987

[B10] Gawin A. Tietze L. Aarstad O. A. Aachmann F. L. Brautaset T. Ertesvåg H. (2020). Functional characterization of three *Azotobacter chroococcum* alginate-modifying enzymes related to the *Azotobacter vinelandii* AlgE mannuronan C-5-epimerase family. *Sci. Rep.* 10:14. 10.1038/s41598-020-68789-3 32719381 PMC7385640

[B11] Gimmestad M. Sletta H. Ertesvåg H. Bakkevig K. Jain S. Suh S. (2003). The *Pseudomonas fluorescens* AlgG protein, but not its mannuronan C-5-epimerase activity, is needed for alginate polymer formation. *J. Bacteriol.* 185 3515–3523. 10.1128/JB.185.12.3515-3523.2003 12775688 PMC156231

[B12] Gorin P. A. J. Spencer J. F. T. (1966). Exocellular alginic acid from *Azotobacter vinelandii*. *Can. J. Chem.* 44 993–998. 10.1139/v66-147

[B13] Govan J. R. Fyfe J. A. Jarman T. R. (1981). Isolation of alginate producing mutants of *Pseudomonas fluorescens*, *Pseudomonas putida* and *Pseudomonas mendocina*. *J. Gen. Microbiol.* 125 217–220. 10.1099/00221287-125-1-217 6801192

[B14] Haug A. Larsen B. (1969). Biosynthesis of alginate. Epimerisation of D-mannuronic acid to L-guluronic acid residues in the polymer chain. *Biochimica et Biophysica Acta* 192 557–559. 10.1016/0304-4165(69)90414-0 5368261

[B15] Haug A. Larsen B. (1971). Biosynthesis of alginate. II. Polymannuronic acid C-5-epimerase from *Azotobacter vinelandii* (Lipman). *Carbohyd. Res.* 2 297–308. 10.1016/s0008-6215(00)82537-9 5150892

[B16] Hay I. D. Rehman Z. U. Moradali M. F. Wang Y. J. Rehm B. H. A. (2013). Microbial alginate production, modification and its applications. *Microbial. Biotechnol.* 6 637–650. 10.1111/1751-7915.12076 24034361 PMC3815931

[B17] Høidal H. K. Ertesvåg H. Skjåk-Bræk G. Stokke B. T. Valla S. (1999). The recombinant *Azotobacter vinelandii* mannuronan C-5-epimerase AlgE4 epimerizes alginate by a nonrandom attack mechanism. *J. Biol. Chem.* 274 12316–12322. 10.1074/jbc.274.18.12316 10212201

[B18] Høidal H. K. Svanem B. I. G. Gimmestad M. Valla S. (2000). Mannuronan C-5 epimerases and cellular differentiation of *Azotobacter vinelandii*. *Environ. Microbiol.* 2 27–38. 10.1046/j.1462-2920.2000.00074.x 11243259

[B19] Klabunde J. Kleebank S. Piontek M. Hollenberg C. P. Hellwig S. Degelmann A. (2007). Increase of calnexin gene dosage boosts the secretion of heterologous proteins by *Hansenula polymorpha*. *FEMS. Yeast Res.* 7 1168–1180. 10.1111/j.1567-1364.2007.00271.x 17617219 PMC2040192

[B20] Linker A. Jones R. S. (1964). A polysaccharide resembling alginic acid from a *Pseudomonas* micro-organism. *Nature* 204 187–188. 10.1038/204187a0 14222269

[B21] Manfrao-Netto J. H. C. Gomes A. M. V. Parachin N. S. (2019). Advances in using *Hansenula polymorpha* as chassis for recombinant protein production. *Front. Bioeng. Biotechnol.* 7:13. 10.3389/fbioe.2019.00094 31119131 PMC6504786

[B22] Mørch Y. A. Donati I. Strand B. L. Skjåk-Bræk G. (2007). Molecular engineering as an approach to design new functional properties of alginate. *Biomacromolecules* 8 2809–2814. 10.1021/bm700502b 17696472

[B23] Moussa M. Ibrahim M. El Ghazaly M. Rohde J. Gnoth S. Anton A. (2012). Expression of recombinant staphylokinase in the methylotrophic yeast *Hansenula polymorpha*. *BMC Biotechnol.* 12:13. 10.1186/1472-6750-12-96 23253823 PMC3539880

[B24] Ramezani-Rad M. Hollenberg C. P. Lauber J. Wedler H. Griess E. Wagner C. (2003). The *Hansenula polymorpha* (strain CBS4732) genome sequencing and analysis. *Fems. Yeast Res.* 4 207–215. 10.1016/S1567-1356(03)00125-9 14613885

[B25] Ramstad M. V. Ellingsen T. E. Josefsen K. D. Hoidal H. K. Valla S. Skjak-Braek G. (1999). Properties and action pattern of the recombinant mannuronan C-5-epimerase AlgE2. *Enzy. Microbial. Technol.* 24 636–646. 10.1016/s0141-0229(98)00148-3

[B26] Stanisci A. Aarstad O. A. Tøndervik A. Sletta H. Dypås L. B. Skjåk-Bræk G. (2018). Overall size of mannuronan C5-Epimerases influences their ability to epimerize modified alginates and alginate gels. *Carbohyd. Polym.* 180 256–263. 10.1016/j.carbpol.2017.09.094 29103504

[B27] Svanem B. I. G. Skjåk-Bræk G. Ertesvåg H. Valla S. (1999). Cloning and expression of three new *Azotobacter vinelandii* genes closely related to a previously described gene family encoding mannuronan C-5-epimerases. *J. Bacteriol.* 181 68–77. 10.1128/JB.181.1.68-77.1999 9864314 PMC103533

[B28] Toikkanen J. H. Sundqvist L. Keranen S. (2004). *Kluyveromyces lactis* SSOI and SEBI genes are functional in *Saccharamyces cerevisiae* and enhance production of secreted proteins when overexpressed. *Yeast* 21 1045–1055. 10.1002/yea.1151 15449305

[B29] Tøndervik A. Aarstad O. A. Aune R. Maleki S. Rye P. D. Dessen A. (2020). Exploiting mannuronan C-5 epimerases in commercial alginate production. *Mar. Drugs* 18:14. 10.3390/md18110565 33218095 PMC7698916

[B30] Tøndervik A. Klinkenberg G. Aachmann F. L. Svanem B. I. G. Ertesvåg H. Ellingsen T. E. (2013). Mannuronan C-5 epimerases suited for tailoring of specific alginate structures obtained by high-throughput screening of an epimerase mutant library. *Biomacromolecules* 14 2657–2666. 10.1021/bm4005194 23808543

[B31] Tøndervik A. Klinkenberg G. Aarstad O. A. Drabløs F. Ertesvåg H. Ellingsen T. E. (2010). Isolation of mutant alginate lyases with cleavage specificity for di-guluronic acid linkages. *J. Biol. Chem.* 285 35284–35292. 10.1074/jbc.M110.162800 20826807 PMC2975152

