# Supplementary material for: Strain Construction and Process Development for Efficient Recombinant Production of Mannuronan C-5 Epimerases in Hansenula polymorpha
Source: Front Plant Sci. 2022 Jun 6;13:837891. doi: 10.3389/fpls.2022.837891 (PMC9208277; doi:10.3389/fpls.2022.837891)
Supplement: Supplementary file 1 [file Data_Sheet_1.docx]

Supplementary file

Comparison of the nucleotide sequence of the original *algE1* and the codon optimized version. Only the changed bases in the codon optimized version are shown.

AvalgE1 catggattacaacgtcaaggatttcggagcactgggcgatggcgtcagcgacgacacggcggccatccag

HPalgE1 ----..c........g.....c.....t..t.....t..c..t..ttcg........c..c..t..t...

AvalgE1 gcggcgatcgacgccgcccacgcggcgggcggcggcaccgtctacctgccggccggcgaatatcgggtca

HPalgE1 ..c..t.................t..c..t..t..a.....t........a.....a..g..ca.a..tt

AvalgE1 gcggcggcgaggagccttccgatggttgtctgaccatcaagagcaacgtccatatcgtcggcgccgggat

HPalgE1 ct..t..t........a..g..c......t...........tcg.....g..c..t..t..t.....t..

AvalgE1 gggcgagacggtgatcaagatggtcgacggctggacgcagaacgtcaccggcatggtgcgctcggcctac

HPalgE1 ...t.....c..............t.....t.....c........t.....t.....ta.a..t......

AvalgE1 ggcgaggaaaccagcaacttcggcatgagcgacctgaccctcgacggcaaccgcgacaacctgtccgcca

HPalgE1 ..t.....g...tcg........a...tcg...t.......g.....t...a.a...........t....

AvalgE1 aggtcgacggctggttcaacggctacatccccggccaggacggcgccgatcgcgacgtgaccctggagcg

HPalgE1 ....t.....a...........t........a..t........t.....ca.a..t..t.........a.

AvalgE1 ggtggaaatccgcgagatgtccggctacggtttcgacccccacgagcagaccatcaacctgacgatccgc

HPalgE1 a.....g...a.a........t..t..............t..................t....c...a.a

AvalgE1 gacagcgtggcccacgacaacagcctcgacggcttcgtcgccgactaccaggtcggcggggtgttcgaga

HPalgE1 ...tcg..t............tct..g.....t.....t..............t..a..a..........

AvalgE1 acaacgtctcgtacaacaacgaccgccacggcttcaacatcgtcaccagcaccaacgacttcgtcctgag

HPalgE1 .......g..t............a.a.....t...........g...tct..............g...tc

AvalgE1 caacaacgtcgcctacggcaacggcggcgccggcctggtggtgcagcgcggctcgtacgacctgccccat

HPalgE1 g........t........t.....t..t..t..t.....t..t...a.a..a..t...........a..c

AvalgE1 ccctacgacatcctgatcgacggcggcgcctactacgacaacgccttggaaggcgtgcagctcaagatgg

HPalgE1 ..a.........t....t.....t..t..................c....g..a..t.....g.......

AvalgE1 cccacgacgtcaccctgcagaacgccgagatctacggcaacggcctgtacggggtgcgcgtctacggcgc

HPalgE1 ..........t..........................a.....a........t...a.a..t.....t..

AvalgE1 ccaggacgtgcagatcctcgacaaccagatccacgacaattcgcagaacggcgcctatgccgaagtcctg

HPalgE1 .........t........g...........t........cagc........t.....c.....g..t...

AvalgE1 ctgcagtcctacgacgacaccgccggggtgtccggcaacttttacgtcaccaccggcacctggctcgaag

HPalgE1 ........t...........t.....t..t..t..t.....c.....g........a........g..g.

AvalgE1 gcaacgtcatcagcggctcggccaattccacctacggcatccaggagcgcgccgacggcaccgactacag

HPalgE1 .a.....t...tct..t..t.....c..t........t.........a.a........a.........tc

AvalgE1 cagcctctacgccaacagcatcgacggtgtgcagaccggggcggtacggctgtatggcgccaactcgacg

HPalgE1 ttct..g.........tcg...........t........t..c..ta.at....c..t...........c

AvalgE1 gtttccagccagtccggcagtggccagcaggcgaccctcgaaggcagcgcgggcaacgatgcgctgagcg

HPalgE1 .....ttct.....t..ttc...t........t.....g..g..ttct..c..t.....c..c...tct.

AvalgE1 ggaccgaggcccacgagacgctgctcggccaggccggcgacgaccgcctgaacggcgatgccggcaacga

HPalgE1 .a.................c...t.g..t.....t..t.....ta.a..t.....t..c.....a.....

AvalgE1 catcctcgacggcggggcagggcgcgacaacctgaccggcggcgcgggcgccgacaccttccgcttctcc

HPalgE1 ...t..g.....a..t..c..ta.a......t.......t..a..t..t............a.a.....t

AvalgE1 gcgcgcaccgacagctaccgcaccgacagcgccagcttcaacgacctgatcaccgacttcgacgccgacg

HPalgE1 ..ta.a......tct...a.a......tct...tct...........t...........t..........

AvalgE1 aggacagcatcgacctgtccgcgctgggcttcaccggcctgggcgacggctacaatggcaccctgctgct

HPalgE1 .....tct...........t..t.....t........tt....t.....a.....c..a...........

AvalgE1 gaagaccaacgccgagggtacgcgcacctacctgaagagctacgaagcggacgcccagggccggcgcttc

HPalgE1 t.................a..ca.a......t.....tct.....g..c...........ta.aa.a...

AvalgE1 gagatcgccctggacggcaacttcaccggtctgttcaacgacaacaacctgttgttcgacgccgctccgg

HPalgE1 ........t........a...........a.....................c..........t..c..a.

AvalgE1 ccaccggtaccgagggcagcgacaacctgctcggcaccgacgccggggaaaccctcctgggctacggcgg

HPalgE1 ..........t.....ttcg.........t.g..a........t..t..g...t.g.....t.....t..

AvalgE1 caacgacaccctcaacggcggggccggcgacgacatcctggtcggcggcgccgggcgcgacagcctgacc

HPalgE1 a...........t.....t..a..a........t........t.....a..t..ta.a...tct..t...

AvalgE1 ggcggcgccggggcggacgtgttccgcttcgacgcgctgtccgacagccagcgcaactacaccaccggcg

HPalgE1 ..a..t..a..a..c.....t...a.a........t.....g...tct...a.a..............t.

AvalgE1 acaaccaggccgaccgcattctcgacttcgacccgaccctggacaggatcgacgtgtcggcgctgggctt

HPalgE1 .............ta.a.....t...........a...........a..t.....t..t..ct....a..

AvalgE1 caccgggctgggcaacggccgcaacggcaccctcgccgtggtgctcaacagcgccggcgaccgcaccgat

HPalgE1 ...t..a.....a.....ta.a.....a...t.g........t..g...tct.....t...a.a..g..c

AvalgE1 ctgaagagctacgacaccgacgccaacggctacagcttcgagctttccctcgcgggcaactaccaggggc

HPalgE1 .............................t...tct......t.g..t..g..t..t...........t.

AvalgE1 agctcagcgccgagcagttcgttttcgcgacgtctcaggggggacagatgacgattatcgaaggcaccga

HPalgE1 ....gtct....................c..c........t..t........c..c..t..g........

AvalgE1 cggcaacgataccttgcagggcaccgaggccaacgagcggctcctcggcctggacggccgggacaacctg

HPalgE1 ...t.....c...........t..t............a.at.g..g..t.........a.a..t..t...

AvalgE1 aacggcggcgccggcgacgacatcctcgacggcggagcggggcgcgacaccctgaccggcggcacggggg

HPalgE1 ..t..a..t..t.....t........t..t..t..c.....aa.a........c..t..a..a..c..a.

AvalgE1 ccgacaccttcctgttctccacgcgtaccgacagctaccgcaccgacagcgccagcttcaacgacctgat

HPalgE1 .a..............t.....ca.a...........ta.a..g........ttcg........tt....

AvalgE1 caccgacttcgatcccacccaggaccgcatcgacctgtccggcctgggcttcagcggtttcggcaacggc

HPalgE1 t..g...........t..t..a..ta.a.........ag...t.....a..ttct..............t

AvalgE1 tacgacggcaccctgctgctgcaggtcaacgccgcgggcacccgcacctacctgaagagtttcgaggccg

HPalgE1 ........t..t...t..t.......t.....t..t..t..ta.a........c..atcc..t.......

AvalgE1 atgccaacggccagcgcttcgagatcgccctggacggcgacttcagcggccaattggacagcggcaacgt

HPalgE1 ..........a...a.a..t.....t...t....t..t......tct..t..gc.....tct..t.....

AvalgE1 gatcttcgagcccgccgtgttcaatgccaaggacttcggcgcgctgggcgacggcgccagcgacgaccgg

HPalgE1 t...........a.....t.....c...........t..a..ct....a..t..t..atc.......a.a

AvalgE1 ccggccatccaggcggcgatcgacgccgcctacgcggccggtggcggcaccgtctacctgccggccggcg

HPalgE1 ..a.....t.....t..c..t..t..t..t.....c..t..a..a..t..t..g...t....a..t..t.

AvalgE1 agtaccgggtcagccccaccggggagccgggcgacggctgcctgatgctcaaggacggcgtctacctggc

HPalgE1 .a...a....gtcg..a.....t.....a..t.....t..t..t.....g........t..t........

AvalgE1 cggcgacggcataggcgaaacggtcatcaagctgatcgacggctccgaccagaagatcaccggcatggtg

HPalgE1 ...t.....a..t..a..g..t...........c........a..g..............t..a......

AvalgE1 cgctcggcctatggcgaagagaccagcaacttcggcatgagcgacctgaccctcgacggcaaccgcgaca

HPalgE1 a.aagc..t..c..a..g..a...........t..t...........t...t.g..t.....ta.a..t.

AvalgE1 acaccagcggcaaggtcgacggctggttcaacggctacatccccggccaggacggcgccgaccgcaacgt

HPalgE1 .....tct..t.....g...........t.....a.....t..t..a..a..t..a..a...a.a.....

AvalgE1 gaccatcgagcgggtggaaatccgcgagatgtccggctatggcttcgatccgcacgagcagaccatcaac

HPalgE1 t..g.....aa.a..c..g..ta.g..a...ag...a..c..a..t.....a.....a..a.....t...

AvalgE1 ctgacgatccgcgacagcgtggcccacgacaacggcctcgacggcttcgtcgccgactacctggtcgaca

HPalgE1 ..c.....ta.a...tct.....t..t..t.....tt.g.....a..t..g..a..t........g...t

AvalgE1 gcgtgttcgagaacaacgtcgcctacaacaacgaccgccacggcttcaacatcgtcaccagcacctacga

HPalgE1 ct..c..t..a...........g..t.....t..ta.g..t.....t.....t.................

AvalgE1 tttcgtcatgaccaacaacgtcgcctacggcaacggcggcgccggcctgacgatccagcggggctcggag

HPalgE1 c..t...........t.....g..t.....a..t..a..a.....t..t..c.....aa.a..t..t...

AvalgE1 gacctggcccagccgaccgatatcctgatcgacggcggcgcctactacgacaacgccctggaaggcgtgc

HPalgE1 ........t.....a.....c.....t........a.....t.....t..t.....t..c.....t....

AvalgE1 tgttcaagatgaccaacaacgtcaccctgcagaacgccgagatctacggcaacggctcctccggcgtgcg

HPalgE1 .............g........g...t.......t..t..a.....t..t.....t..t..t..t..ca.

AvalgE1 cctgtacggcacggaggacgtgcagatcctcgacaaccagatccacgacaattcgcagaacggcacctat

HPalgE1 a........a..t..............tt.g..t..t..a........t..c...........a.....c

AvalgE1 ccggaagtcctgctgcaggccttcgacgacagccaggtcaccggtgagctgtacgagaccctgaacaccc

HPalgE1 ..a.....gt.............t......tct.....t.....a...............t........a

AvalgE1 ggatcgaaggcaatctcatcgacgcttcggacaacgccaactatgcggtgcgcgagcgcgacgacggcag

HPalgE1 .a.....g..a..ct.g........c.................c..c..ta.a..aa.a..t.....ttc

AvalgE1 cgactacaccacgctcgtggacaacgacatcagcggcggccaggtcgcctcggtgcagctttccggcgcc

HPalgE1 g........t..c..t..............ttcg..t..t.....t..a..t........g..t..t...

AvalgE1 cattcgagtctttccggcggcaccgtcgaagtgccgcaggggaccgacggcaacgacgtgctggtcggca

HPalgE1 ..c..ttc.t.g..t..t........t..g..t..a.....t..a.....a..t...........t..at

AvalgE1 gcgatgccaacgaccagctctacggcggagccggcgacgaccgcctggacggcggcgccggtgacgacct

HPalgE1 cg..c............t.g.....a..c..a..t......a.at....t..t..a.....a..t.....

AvalgE1 gctcgacggcggagcggggcgcgacgacctgaccggcggcacgggtgccgacaccttcgtgttcgccgcg

HPalgE1 .t.g..t.....t..a..aa.g..t.....t.....t.....c..c..a..t..t.....t..t.....a

AvalgE1 cgtaccgatagctaccgcaccgacgcgggggtgttcaacgacctgatcctcgacttcgacgccagcgagg

HPalgE1 a.a..g...tcg..ta.a..a.....c..t..t.....t.....c.....g............tca....

AvalgE1 accgcatcgacctgtccgccctgggtttcagcggcttcggcgacggctacaacggcaccctgctggtgca

HPalgE1 ..a.a......t....g..a..c..a...tct..a..t..t..t..t.....t......t..........

AvalgE1 gctcagcagcgccggaacccgtacctacctcaagagctacgaggaggacctcgagggccggcgcttcgag

HPalgE1 ...ttcttcg.....t..ga.a..t..t..g...tcg..t...........g.....aa.aa.a..t..a

AvalgE1 gtcgccctggacggcgaccacacgggcgatctttccgccgccaatgtggttttcgccgacgacggctcgg

HPalgE1 ..g..at.......a........c..t..ct.g..t........c..t.....t..a........t..t.

AvalgE1 ccgccgtggcgagcagcgatcccgccgccacacagttggaggtggtcggcagcagcggcacccagaccga

HPalgE1 .......t..ctcttcg..c..a........c...c.......t..g..atcttct..a.....a.....

AvalgE1 tcaactcgcctga

HPalgE1 c..g..g....a.

Comparison of the nucleotide sequence of the original *algE4* and the codon optimized version. Only the changed bases in the codon optimized version are shown.

AvalgE4 atggattacaacgtcaaggatttcggtgcattgggcgacggcgtcagcgacgaccgggcc

HpalgE4 ---..c...........a..c........t.....t.....t..ttct......a.a...

AvalgE4 tccatccaggcggcgatcgatgccgcctacgccgccggtggcggtaccgtctacctgccg

HpalgE4 ..t..t.....t..t.....c..t..t.....t..a.....t..a..t..t...t....a

AvalgE4 gccggcgagtaccgggtcagcgccgccggggagccgggcgacggctgcctgatgctcaag

HpalgE4 .....t......a.a..ttct..t..t..c.....a..t.....t..t........g..a

AvalgE4 gacggcgtctacctggccggtgccggcatgggcgagacggtgatcaagctgatcgacggc

HpalgE4 .....t..t...t....t.....t..a...........c..t.................t

AvalgE4 tccgaccagaagatcaccggcatggtccgctcggcctacggcgaggaaaccagcaacttc

HpalgE4 ..t........a........a.....ga.a..t..t...........g...tct......

AvalgE4 ggcatgcgcgacctgaccctcgacggcaaccgcgacaacaccagcggcaaggtcgacggc

HpalgE4 ..a...a.a...t.......g.....t...a.a.........tct..a.....t.....t

AvalgE4 tggttcaacggctatatccccggcggggacggcgccgaccgcgacgtgaccatcgagcgg

HpalgE4 ...........t..c.....a..t..t.....t..t...a.a..t..t.........a.a

AvalgE4 gtggaggtccgcgagatgtccggctacggcttcgacccccacgagcagaccatcaacctg

HpalgE4 ..t..a...a.a........t..t.....t..t.....a..............t......

AvalgE4 acgatccgcgacagcgtggcccacgacaacggcctcgacggcttcgtcgccgactacctg

HpalgE4 ..c...a.a...tct..t..t...........a..g.....t..t..t............

AvalgE4 gtcgacagcgtgttcgagaacaacgtcgcctacgccaacgaccgccacggcttcaacgtg

HpalgE4 ..g...tct.................t........t......a.a.....t.........

AvalgE4 gtcaccagcacccacgatttcgtcatgaccaacaacgtcgcctacggcaacggcagcagc

HpalgE4 ..t...tct........c.....g..............g..t.....t.....ttcttct

AvalgE4 ggcctggtggtgcagcggggtctggaggacctcgcgctgcccagcaacatcctgatcgac

HpalgE4 ..a.....t......a.a.....t......t.g..t.....atct...........t...

AvalgE4 ggcggcgcctactacgacaacgcccgcgaaggcgtgctgctcaagatgaccagcgacatc

HpalgE4 ..t..t..t...............a.a..g..t..t..t..g.........tct......

AvalgE4 accctgcagaacgccgatatccacggcaacggctcctccggggtgcgcgtctacggcgcc

HpalgE4 ...t..........t..c..t.....a.....t.....t..t..ta.a..t.....t...

AvalgE4 caggacgtgcagatcctcgataaccagatccacgacaacgcgcaggcggccgccgtgccc

HpalgE4 ........t.....t..g..c....................t.....t..t..t..t..a

AvalgE4 gaggtcctgctgcagtccttcgacgataccgccggggcgtccggcacctactacacgacc

HpalgE4 .....t...........t..t.....c..t..t..t..a..t..t..t........c...

AvalgE4 ctgaacacccggatcgagggcaacaccatcagcggctcggccaactccacctacggcatc

HpalgE4 .........a.a..t.....t.........tct..t..t........t........t...

AvalgE4 caggagcgcaacgacggcaccgactacagcagcctgatcgacaacgacatcgccggggtg

HpalgE4 ......a.a........t..t......tcttct.................t..t..t..t

AvalgE4 caacagcccatccaactgtacggacctcactcgacggtatccggcgaacccggcgcgaca

HpalgE4 ..g.....a..t..gt.......t..a.....t..t..t..t.....g..t..t..t..t

AvalgE4 ccgcaacagccgtccacgggaagcgacggcgagccactggtcggcggcgacacggacgac

HpalgE4 ..a..g.....a..t..t...tct............t....t..t..t.....t......

AvalgE4 cagctccagggcggctccggcgccgatcgcctggacggcggggccggcgacgacatcctc

HpalgE4 ...t.g........t..t..t..a..ca.a........t..t..a..t........tt.g

AvalgE4 gacggcggcgccgggcgcgaccggctgagcggcggcgcgggcgccgacaccttcgtgttc

HpalgE4 .....t.....t..ta.a...a.a..ttct..t..t..t.....t...........t...

AvalgE4 tccgcccgcgaggacagctaccgtaccgacacggcggtgttcaacgacctgatcctcgac

HpalgE4 ..t...a.a......tct...a.a........c..c..t.................g...

AvalgE4 ttcgaggccagcgaggatcgcatcgacctgtccgcgctgggcttttccggcctgggcgac

HpalgE4 ..t.....ttct.....ca.a..t........t..c.....a.....t..t..t..t...

AvalgE4 ggctatggcggcaccctgctcctgaagaccaacgccgagggcacgcgcacctacctgaaa

HpalgE4 ..t..c..t..a...t....gt....a........t.....a..ca.a...........g

AvalgE4 agcttcgaggcggatgccgagggacggcgcttcgaggtcgccctggacggcgaccacacg

HpalgE4 tct........t..c..t.....ta.aa.a..t.....t...t.......t........t

AvalgE4 ggcgatctttccgccgccaatgtggtcttcgccgcgaccgggacgaccaccgaactcgaa

HpalgE4 ..t..ct.g..t........c..t..t..t..t..t.....t..t..t.....g..t..g

AvalgE4 gtgctcggcgacagcggcacgcaggccggggcgatcgtctag

HpalgE4 ..tt.g......tct..t..t.....t..t..c.....g..a

Comparison of the nucleotide sequence of the original *algE6* and the codon optimized version. Only the changed bases in the codon optimized version are shown.

AvalgE6 atggattacaacgtcaaggatttcggagcactgggcgacggcgtcagcgacgaccgggtc

HpalgE6 ---..c........t.....c.....t..tt....t.....t..ttct......a.a..t

AvalgE6 gccatccaggcggcgatcgacgccgctcacgccgcgggcggcggtaccgtctacctgccg

HpalgE6 .....t.....t..t..t.....t........t..c..t..t.....t..t...t....a

AvalgE6 cccggcgaataccgggtcagcgccgccggggaaccttccgacggctgcctgacgctcagg

HpalgE6 ..a.....g...a.a..ttct..t..t..c..g..a..t.....t..tt....c..g..a

AvalgE6 gacaacgtctacctggccggcgccggcatgggccagacggtgatcaaactggtcgacggc

HpalgE6 ........t...t.......t..t..a.....a.....c..............t.....t

AvalgE6 tcggcccagaagatcaccggcatagtccgctcgcccttcggcgaggaaaccagcaacttc

HpalgE6 ..t....................t..ta.a..t..a........a..g..ttct......

AvalgE6 ggcatgcgcgacctgaccctggacggcaaccgcgccaacaccgtcgacaaggtcgacggc

HpalgE6 ......a.a...t....t........t...a.a..t........g.....a..g.....t

AvalgE6 tggttcaacggctatgcccccggccagccgggcgccgatcgcaacgtgaccatcgagcgg

HpalgE6 ...........t..c..t..a..a.....a..t..t..ca.a...............a.a

AvalgE6 gtggaggtccgcgagatgtccggctacggcttcgacccccacgagcagaccatcaacctg

HpalgE6 ..t..a..ta.a........t........t..t.....a..............t......

AvalgE6 gtgctgcgcgacagcgtggcccatcacaacggcctcgacggcttcgtcgccgactaccag

HpalgE6 ......a.a...tct..t..t..c........a..g.....t..t..t............

AvalgE6 atcggcggaaccttcgagaacaacgtcgcctacgccaacgaccgccacggcttcaacatc

HpalgE6 ..t..t....................t........t......a.a.....t.........

AvalgE6 gtcaccagcaccaacgacttcgtcatgcgcaacaacgtcgcctacggcaacggcggcaac

HpalgE6 ..g...tcg..............g...a.a........g..t...........t..t...

AvalgE6 ggcctggtggtgcagcggggttcggaaaacctcgcccatcccgagaatatcctgatcgac

HpalgE6 ..a.....t......a.a.....t..g...t.g..t..c..a.....c..t.........

AvalgE6 ggcggctcctactacgacaacggcctggaaggcgtgctggtcaagatgagcaacaacgtc

HpalgE6 .....t..t...............t....g..t..t.....g......tcg........g

AvalgE6 accgtgcagaacgccgatatccacggcaacggctcctccggggtgcgcgtctacggcgcc

HpalgE6 .....t........t..c..t..t..t..t..t..t..g..t..ta.a..t.....t...

AvalgE6 cagggcgtgcagatcctcggcaaccagatccacgacaacgcgaagacggccgtcgccccg

HpalgE6 .....t..t.....t..g..t....................t.....t..t..t..t..a

AvalgE6 gaagtgctgctgcagtcctacgacgataccctcggcgtgtccggcaactactacacgacc

HpalgE6 ..g..t...t.......t........c...t.g..t.....t..t...........c...

AvalgE6 ctgaacacccgggtcgagggcaacaccatcaccggctcggccaactccacctacggcgtg

HpalgE6 ........ta.a..t..............t.....t..t........t..t.........

AvalgE6 caggagcgcaacgacggcaccgacttcagcagcctggtcggcaacaccatcaacggcgtg

HpalgE6 ..a...a.a........a.........tcttct.....g..t..............t..t

AvalgE6 caggaggctgcccacctgtacggtccgaattcgacggtctccggcactgtcagcgcgccg

HpalgE6 ..a........t...t..........a..c..t..c..t..t..a..c..ttct..t..a

AvalgE6 ccgcaagggaccgacggcaacgacgtgctgatcggcagcgacgtcggcgagcagatcagc

HpalgE6 ..a..g..t..t.....t..............t..ttct..t..g..a........ttct

AvalgE6 ggcggagccggcgacgatcgcctggacggcggggccggcgacgacctgctcgacggcggc

HpalgE6 ..t..t..t..t.....ca.at.......a..a..a..t..t..tt..t.g..t..t..a

AvalgE6 gccgggcgcgaccggctgaccggcggcctgggcgccgacaccttccgcttcgccctgcgc

HpalgE6 ..t..aa.a..ta.a.....t..t..at....t..t.........a.a..t...t..a.a

AvalgE6 gaggacagccatcgctcgccgctgggcaccttcagcgacctgatcctcgacttcgatccg

HpalgE6 ......tct..ca.a..t..a.....a......tct........t..g.....t..c..a

AvalgE6 agccaggacaagatcgacgtgtcggcgctcggtttcatcggcctgggcaacggctacgcc

HpalgE6 tct...........t.....t..t..c..g.....t..t..a.....t.....a..t..t

AvalgE6 ggcaccctggcggtgagcctcagcgccgacggcctgcgcacctacctgaagagctacgac

HpalgE6 ..t..t.....c..ttctt.gtct..t.....a...a.a...........atct......

AvalgE6 gcggacgcccagggccgctccttcgaactggcgctggacggcaaccatgccgcgaccctg

HpalgE6 ..t.....t.....aa.a..t..t..g.....tt.......t..t..c..t..t..a...

AvalgE6 tcggcgggcaacatcgtcttcgccgcggccaccccggtcgaccccagcgccgaggcgcaa

HpalgE6 ..t..c..t.....t..t..t.....c..t..t..a..t..t..atct........t...

AvalgE6 ccgatcgtcggcagcgacctcgacgaccagttgcacggcaccctgctcggcgaggagatc

HpalgE6 ..a..t..t..ttct.....g........a.....t..t..t.....g..t..a..a..t

AvalgE6 agcggcggcggcggcgccgaccaattgtacggctatggcggcggcgacctgctcgacggc

HpalgE6 tct..t..a..t..t..t..t...........t..c..t..t..t..t..c..t.....t

AvalgE6 ggcgccgggcgcgaccggctgaccggcggcgagggcgccgacaccttccgcttcgccctg

HpalgE6 ..t..a..aa.a...a.at....a.....t..a..t........t..ta.a.....t..c

AvalgE6 cgcgaggacagccaccgctcggcggcgggcaccttcagcgacctgatcctcgacttcgat

HpalgE6 a.a...........ta.a..t..t..t..t..t..ttcg...........t........c

AvalgE6 ccgacccaggacaagctcgacgtatcggcgcttggcttcaccggcctgggcaacggctac

HpalgE6 ..t.....a........g..t..t..t..tt.g..t........at....a..t..t..t

AvalgE6 gccggcaccctggcggtgagcgtcagcgacgacggcacgcgcacctacctgaagagctac

HpalgE6 ..g..a...t....t..ttct..gtct........a..ca.a..g...t.....tct...

AvalgE6 gagacggatgccgagggccgctccttcgaggtcagcctgcagggcaaccatgccgccgcg

HpalgE6 .....a.....t......a.a..t.....a..ttct........t........t..t..t

AvalgE6 ctgtcggccgacaacatcctgttcgccacgcccgtgccggtcgatcccggcgtcgaggga

HpalgE6 .....t..t........t...........t..a..t..a..t..c..a..t..t.....t

AvalgE6 acgccggtggtcggcagcgacctcgacgacgagttgcacggcaccctgggcagcgagcag

HpalgE6 ..t..a..t..g..ttct...t.g..t..t...c.......t..tt....ttct......

AvalgE6 atcctcggcggcggcggcgccgaccagttgtacggctacgccggcaacgacctgctcgac

HpalgE6 ..tt.g..a..t..t..t..t.....ac.t..t..a........t.....t..t..t..t

AvalgE6 ggcggcgccggtcgcgacaagctgagcggcggcgagggcgccgacaccttccgcttcgcc

HpalgE6 .....t..t...a.a..t..at..tct.....a........t..t..t...a.a.....g

AvalgE6 ctgcgcgaggacagccaccgctcgccgttggggaccttcggcgaccgcatcctcgacttc

HpalgE6 ...a.a..a..ttct..ta.aagc..a.....a.....t..t...a.a.....g......

AvalgE6 gatccgagccaggacaggatcgacgtctcggcgctgggcttttccggcctgggcaacggc

HpalgE6 .....ttct.....t..a........g..t..t.....a.....t...t....t.....t

AvalgE6 tatgccggtagcctggcggtgagcgtcagcgacgacggcacgcgcacctacctgaagagc

HpalgE6 ..c..a..atct..t..c..ttcg..ttct.....t......a.a..t.....t......

AvalgE6 tacgaggcggacgcccagggtctgagcttcgaggtggccctggagggcgaccatgccgcc

HpalgE6 ........a.....a......t..tct..t.....t..at.............c..t..a

AvalgE6 gcgctgtcggccgacaacatcgtcttcgccgcgaccgacgctgcggcggccggcgaactc

HpalgE6 ..at..agc..t........t..t........t..t........t..a..g..a..gt.g

AvalgE6 ggggtgataggcgccagcggccagccggacgatccggccgtctga

HpalgE6 ..t..t..t..t..ttct..a.....a.....c..a.....t.a.
